# Current evidence on the effectiveness of Ready-to-Use Supplementary Foods in children with moderate acute malnutrition: a systematic review and meta-analysis

**DOI:** 10.1017/jns.2023.114

**Published:** 2024-01-03

**Authors:** Melese Sinaga Teshome, Tefera Belachew Lema, Teklu Gemechu Abessa, Sarah Mingels, Marita Granitzer, Eugene Rameckers, Evi Verbecque

**Affiliations:** 1 Department of Nutrition and Dietetics, Faculty of Public Health, Health Institute, Jimma University, Jimma, Ethiopia.; 2 Rehabilitation Research Centre (REVAL), Faculty of Rehabilitation Sciences, Hasselt University, 3590 Diepenbeek, Belgium; 3 Department of Special Needs and Inclusive Education, Jimma University, Jimma, Ethiopia; 4 Musculoskeletal Research Unit, Department of Rehabilitation Sciences, Faculty of Kinesiology and Rehabilitation Sciences, Leuven University, Leuven 3000, Belgium; 5 Research School CAPHRI, Department of Rehabilitation Medicine, Maastricht University, Maastricht, The Netherlands; 6 Centre of Expertise in Rehabilitation and Audiology, Hoensbroek, The Netherlands

**Keywords:** Children, Moderate acute malnutrition, Ready-to-Use Supplementary Food, RUSF, School-age children, Wasting

## Abstract

Moderate acute malnutrition (MAM) is defined by a weight-for-height *Z*-score (WHZ) between −3 and −2 of the WHO reference or by a mid-upper arm circumference (MUAC) of ≥11⋅5 and <12⋅5 cm. This study aimed to synthesise the evidence for the effectiveness of Ready-to-Use Supplementary Food (RUSF) compared to other dietary interventions or no intervention on functioning at different levels of the International Classification of Functioning, Disability, and Health (ICF) among children with MAM between 2 and12 years old. Three databases (PubMed, Scopus, and Web of Science) were systematically searched (last update: 20 November 2022). Pooled estimates of effect were calculated using random-effects meta-analyses. The level of evidence was estimated with the Grading of Recommendations, Assessment, Development, and Evaluation (GRADE) method. Seven studies were included. RUSF had a significant small-sized better effect (pooled mean: 0⋅38; 95 % CI = [0⋅10, 0⋅67], *P* = 0⋅01, *I*² = 97 %) on different anthropometric measurements compared to other dietary interventions among MAM children (*n* 6476). Comparing RUSF with corn–soy blend Plus Plus (CSB++) showed that RUSF had a small-sized but significantly better effect on the children's anthropometric measures compared to children who received CSB++ (pooled mean: 0⋅16; 95 % CI = [0⋅05, 0⋅27], *P* = 0⋅01; *I*^2^ = 35 %). MAM children treated with RUSF had a better recovery rate compared to those treated with CSB++ (pooled risk difference: 0⋅11; 95 % CI = [0⋅06, 0⋅11], *P* < 0⋅001; *I*^2^ = 0 %). The RUSF intervention seems promising in improving MAM children's nutritional outcomes and recovery rate compared to other dietary interventions.

## Introduction

Child undernourishment is a significant public health issue, particularly in many low- and middle-income nations. It indirectly hurts a country's production and presents difficulties on the economic and social fronts for vulnerable populations.^(1,2)^ Poor nutrition is associated with suboptimal brain development, which negatively affects cognitive development, educational performance, and economic productivity in adulthood.^(1)^ Malnourishment in early life hurts the development of language, motor, and cognitive skills in children, and the deficits can be tracked through childhood and into adult life,^(2–4)^ thereby posing a high risk of morbidity and mortality if not treated.^(5)^ Malnourishment is broadly classified as *undernourishment*, which includes wasting (low weight-for-height), underweight (low weight-for-age), stunting (low height-for-age), and micronutrient-related malnutrition, versus *overnourishment*, which includes obesity, overweight, and nutrition-related noncommunicable diseases.^(6)^ One of the groups of undernourished children who deserve more attention is that with moderate acute malnutrition (MAM), which is defined by the World Health Organisation (WHO) as a weight-for-height *Z*-score (WHZ) between −2 and −3 or mid-upper arm circumference (MUAC) between 115 and <125 mm for children under five.^(7)^ For children 5–19 years old, MAM is considered when the BMI for the age *Z*-score is −3 to <−2.^(8)^

Around the world, MAM affects around 45⋅4 million children, 97 % of whom live in LMICs (low- and middle-income countries).^(9,10)^ It disproportionately affects more than 19 million children under the age of 5 years and results in more than 1 million child fatalities annually.^(9)^ By 2020, an additional 140 million children could live in poverty as a result of the COVID-19 pandemic, and 6–7 million more children would suffer from wasting or acute malnutrition.^(11)^

Children suffering from MAM often live in the community because their condition is not life-threatening, in contrast to children with severe acute malnourishment (SAM). However, children with MAM have a threefold greater risk of mortality, an increased risk of infections, and poor physical and cognitive development,^(12)^ that transcend into adolescence and adulthood. For instance, moderate-to-severe acute malnutrition throughout infancy and childhood is linked to a notably higher prevalence of impaired physical growth, motor, and social skill development, more behavioural issues, and even a lower intellectual quotient in adulthood.^(13–15)^ Additionally, wasting throughout adolescence and during the school years might put off pubertal development, the development of muscular power, and potentially even the ability to work.^(16,17)^

Malnutrition slows down the brain's ability to develop quickly by adversely altering its structural and functional capabilities, which causes developmental abnormalities in children across all domains (biological, psychological, and social),^(18,19)^ which in turn further impacts the developmental delay in these children,^(20)^ and at different ages. Even though neurulation and the formation of the receptive language, vision, and hearing brain areas occur during fetal life and before the age of 5 years, synaptogenesis and neurogenesis continue up to the end of adolescence.^(21)^ Peaks of brain development, especially of the frontal lobes, have been found in the first 2 years, 7–9 years, and in the mid-teenage years.^(22,23)^ Most research focuses on the young child (birth to 5 years of age) because of the detrimental impact of malnutrition on their overall functioning. However, older children who suffer from malnutrition deserve attention as well. School-age children have a higher risk of being affected by different nutritional and health problems due to the high energy demand for rapid growth and high engagement in physical activity.^(24)^ This growth spurt significantly raises the amount of nutrients needed. As a result, a child's physical and mental growth during the primary school years depends on a healthy diet, especially if they are already undernourished.^(25)^ Consuming enough energy, protein, calcium, iron, zinc, and folate is essential, especially when development is at its fastest and nutritional needs may be up to twice as high as during the rest of adolescence.^(26,27)^ Due to physical growth and cognitive development, the nutrient requirements of school-aged children are high.^(28)^ Thus, nutrient deficiencies may be especially harmful to cognitive and physical development during school age and early adolescence. School-age and adolescence are also critical periods for neurological development. Therefore, the age range of 2–12 years is a critical period in development, emphasizing the detrimental impact of malnourishment on overall development.

Dietary interventions for MAM aim to rehabilitate these children and prevent further deterioration of nutritionally vulnerable groups and other health-related problems. Ready-to-use therapeutic foods were initially developed to target SAM and served as a basis to develop Ready-to-Use Supplementary food (RUSF) for MAM which have found their way to the MAM children in the past decade.^(29,30)^

So far, several systematic reviews and meta-analyses have been conducted on the effectiveness of dietary interventions on the nutritional status of MAM children less than 5 years old, particularly infants (birth to 2 years of age).^(31,32)^ However, from a developmental and epidemiological point of view, insights into the effects of dietary interventions on toddlers and school-aged children are highly relevant. Furthermore, due to the large impact that malnutrition has beyond nutritional status, a more comprehensive systematic review and meta-analysis looking into the effectiveness of dietary interventions on functioning at different levels of the International Classification of Functioning, Disability, and Health (ICF) among children with MAM is both new and much needed. The present review fills this gap.

This review aims to assess the effectiveness of RUSF compared to any other type of dietary intervention or no intervention on functioning at different levels of the ICF among children with MAM aged between 2 and 12 years old.

## Methods

This systematic review was conducted following the Preferred Reporting Items for Systematic Reviews and Meta-Analyses (updated PRISMA guidelines 2020)^(33)^. The study protocol was registered in PROSPERO (www.crd.york.ac.uk/prospero/display_record.php?Record ID = CRD42022295693).

### Eligibility criteria

The PICOS criteria (population, intervention, comparison, outcome, and study design) were used for study selection. The studies discussing the effectiveness of nutrition therapy or dietary interventions used for the management of MAM in children aged 2–12 years old without medical complications were included for data extraction. More specifically, the following selection criteria were applied:

#### Population

To fulfil the criterion of MAM, the included children had to be diagnosed by the WHO growth standard and reference using MUAC ≥11⋅5 to <12⋅5 cm and/or a weight-for-length/height *Z*-score (WLZ) ≥−3 to <−2, or WFH (weight-for-height) ≥70 to <80 % of the median National Center for Health Statistics (NCHS) growth references, in the absence of bilateral pitting oedema. Children between 2 and 12 years of age were of interest, as literature on infants had been summarised previously.^(32)^ If studies included younger children, the group mean age had to be 24 months or more to be included, as then at least half of the children would be in the age range of interest. Studies on adults, children with SAM, and any other primary condition affecting child development other than malnutrition, i.e. neurological (e.g. cerebral palsy), musculoskeletal disorder (e.g. torticollis), neurodevelopmental disorders (e.g. developmental coordination disorder (DCD), or autism spectrum disorder (ASD), or cardiovascular (e.g. congenital heart failure) disorder, were excluded.

#### Intervention

The study compared RUSF to any type of dietary intervention or no intervention. Non-nutrition interventions, including drug or medical interventions, research reporting on the effects of supplementary feeding in refugee settings, hospitalised patients (after injury, surgery, or other acute medical conditions), enteral tube feeding or parenteral feeding products, and therapeutic feeds for the treatment of SAM were excluded.

#### Outcome

Any domain of the ICF framework, such as the function level (e.g. muscle strength, nutritional status, body composition, linear growth, etc.), activities of daily life (e.g. mobility, dressing, self-care, or play-based activities), or participation (e.g. family, peers, and leisure activities), was included for data extraction.

#### Design and language

All controlled clinical trials, whether or not randomised, were included in this review. Studies with any other design were excluded, such as editorials, conference proceedings, abstracts only, case reports, case series, newspapers, cross-sectional studies, qualitative formative assessments, discussion papers, thesis dissertation papers, and unpublished papers. Any language other than English was excluded from this review. The references to systematic) reviews with the same scope were screened to avoid missing relevant studies.

### Search methods

PROSPERO registrations, as well as databases, were explored to confirm whether previous systematic reviews and/or meta-analyses exist to avoid duplicates. The following electronic databases were searched, MEDLINE through the Pubmed interface, Web of Science, and Scopus. These electronic databases were selected given their well-established relevance to health and their complementarity.

The initial search string was developed in PubMed (MEDLINE) and then adjusted for the other databases (Supplementary Appendix I). The search terms were grouped into the main concepts of ‘MAM’, ‘nutrition therapy’, and ‘children’ aged 2–12 years old. Next, a systematic approach to finding relevant articles was applied by combining Medical Subject Headings (MeSH), keywords, Boolean operators (‘AND’ and ‘OR’), truncation, and field tags. The final search was updated on 20 November 2022. The search was restricted to ‘human studies’. No other filters were applied. Finally, the reference lists of identified studies were also checked for additional papers that met the inclusion criteria.

### Screening and study selection

**Fig. 1.**
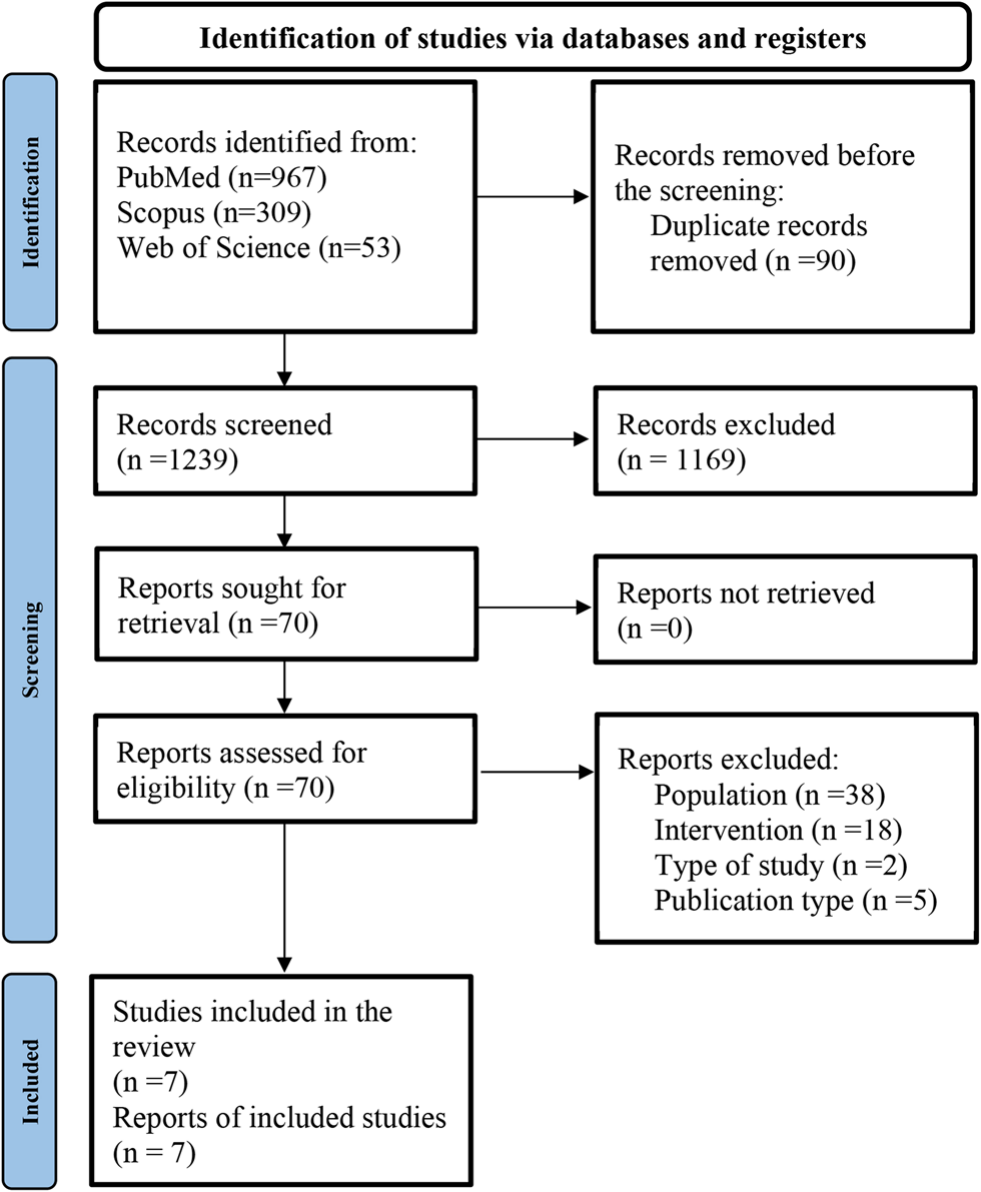
PRISMA flow diagram of the study selection. *N*, number.

All records were screened independently by two reviewers (M.S.T. and E.V). In the first phase, titles and abstracts of all search results were screened, guided by the PICO framework. In the second phase, full texts were evaluated (Fig. 1). Disagreements about the appropriateness of the inclusion of studies were resolved in a consensus meeting through discussion. Records of the reasons for exclusion were kept at title, abstract, or full-text screening.

### Risk of bias assessment of individual studies

Two reviewers (MST and EV) independently assessed the risk of bias for each controlled trial using the criteria outlined in the revised Cochrane Risk of Bias Tool for Randomised Trials (RoB2).^(34)^ The RoB2 considers the following domains: (1) bias arising from the randomisation process; (2) bias due to deviations from the intended intervention; (3) bias due to missing outcome data; (4) bias in the measurement of the outcome; (5) bias in the selection of the reported result and incomplete outcome data; and (6) selective reporting. For each criterion, a risk of bias judgment for each domain was made to estimate the level of bias: ‘low risk of bias, ‘a high risk of bias’, or ‘some concerns of bias’ (Table 1).

**Table 1.** Risk of bias assessment for the seven included studies

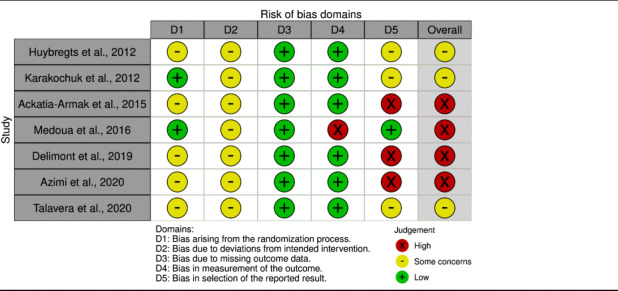

### Data extraction and management

The data were first extracted independently by one reviewer (M.S.T.) and checked by a second reviewer (E.V.). The extraction was done, including details of methods (design and study duration), population (sample size, sex distribution, mean age and sd, age range), interventions (RUSF and any other type of dietary intervention, including Kcal/day and the duration of the treatment), outcomes (any domain of the ICF), study design (RCT and cluster RCT), and results (group means and standard deviations). When study information was missing, the reviewer's team attempted to contact the primary author of the incomplete study. A study was excluded if the author did not respond. Any discrepancies were resolved through discussion. A descriptive synthesis was done to summarise findings about the characteristics of children who recovered from MAM versus those who did not recover from MAM.

### Data analysis

Statistical Package for Social Sciences (SPSS) version 28.0 software was used to perform random-effects meta-analyses to estimate pooled (standardised) mean differences and risk differences for the outcomes. Random-effects meta-analyses were chosen to incorporate the expected random variation in the effect of each intervention across the studies into the pooled estimates.^(35,36)^ Data on continuous outcomes were analysed using standardised mean differences (SMD) because the outcomes were measured with similar, but not identical, instruments across studies. To maximise the data input for the pooled outcome measures, we used the post-intervention values (means and SDs) in preference to the changes from the baseline. Post-intervention data were included in the analysis only if the pre-intervention data were not statistically different between groups. All results are presented with 95 % confidence intervals.

Clinical diversity (variability in the participants, interventions, and outcomes of the studies) and methodological diversity (study design, risk of bias) as well as statistical diversity were considered to assess heterogeneity. The statistical heterogeneity was assessed using a standard *χ*^2^ test and *P*-value to see the strength of evidence for heterogeneity, and we used the *I*^2^ test to evaluate the impact of heterogeneity on the meta-analysis. If the percentage shows the variability in the effect estimate due to heterogeneity rather than by chance, values of 75–100 % indicate considerable heterogeneity.^(35)^ We obtained the estimate between the studies’ variance component (τ^2^) through a random-effects meta-analysis model. In the case of too large heterogeneity, subgroup analysis was considered to account for the clinical diversity in the following sequence: intervention, outcomes, and participants.

### Level of evidence

In studies comparing the RUSF dietary intervention to another dietary supplementation, control, or no intervention group, the evidence of quality was appraised using the Grading of Recommendations, Assessment, Development, and Evaluation (GRADE) method.^(37)^ Study limitations, inconsistency of results, indirectness of evidence, and imprecision were used in assessing the quality of the evidence. Two reviewers (MST and EV) independently assessed the quality of the evidence assessment, and any discrepancies were resolved through discussion.

## Results

### Study selection

A total of 1329 potentially important titles from the different electronic databases were generated. After removing 90 duplicates, a total of 1239 papers were screened, and 1169 papers were excluded based on title and abstract. Finally, 70 studies were selected for more detailed evaluation, seven of which were eligible for inclusion in this systematic review and meta-analysis. Fig. 1 shows the search flow diagram and the reason for the exclusion of the studies.

### Risk bias assessments

Half of the studies had an overall high risk of bias, whereas, for the other half, some concerns were identified (Table 1). All studies showed a low risk of bias regarding the handling of missing data. All, except one, had a low risk of bias in the measurement of the outcome. Some concerns were identified in some studies regarding bias arising from the randomisation process, and in all studies, bias arose from deviations from the intended intervention. The diverse risk of bias was identified in the selection of reported results (three studies showed high risk, four showed some concerns, and one showed low risk).

### Study characteristics

The seven eligible papers included a total of 6476 children. The study characteristics are presented in Table 2. Five studies were conducted in African countries, i.e. two in West Africa (Mali and Cameroon),^(38,39)^ two in East Africa (Tanzania and Ethiopia),^(40,41)^ and one in Central Africa (Chad),^(42)^ and the other two studies were conducted in the Middle East (Iran),^(43)^ and North America (Mexico).^(44)^ Four studies were done in rural areas,^(38–41)^ one in urban areas,^(42)^ and one in both rural and urban settings.^(39)^ Out of seven studies, three of them were randomised controlled trials (RCTs),^(39,43,44)^ and four of them were Cluster-Randomised Controlled trials (cluster-RCTs).^(38,40–42)^ Sample sizes ranged from 81 to 2186.^(39,40)^ Enrolment ages differed (Table 2), and were limited to 6–60 months. All studies defined MAM with either a WLZ based on WHO 2006 Growth Standards and/or a MUAC. The treatment duration varied between 8 and 20 weeks. The characteristics of the included studies are illustrated in Table 2.

**Table 2.** Characteristics of the included studies (methods, participants, interventions, and outcomes)

Reference	Methods		Participants			Intervention(s)/Control			Outcomes	
	Study design	Study duration (weeks)	Country (rural/urban/both)	*n* (males)	Age in months	Types of intervention	Kcal/day,	Study design	Outcome measures	ICF level (F/A/P)
Ackatia-Armah, 2015	Cluster RCT	12	Mali (rural)	1264 (592)	6–35 months	CSB++ *v.* LMF *v.* MI *v.* RUSF.	500 kcal	Community-based	Weight gain, MUAC, length, WLZ, LAZ, haemoglobin level, % anaemia, % recovery	F
Azimi, 2020	RCT	8	Tehran, Iran (urban)	100 (49)	24–59 months	RUSF *v.* control	75 kcal/kg/day,	Facility-based	Weight gain, length, BMI, WAZ, % recovery	F
Delimont, 2019	Cluster RCT	20	Tanzania (rural)	2186 (409)	6–53 months	WSC1 *v.* WSC2 *v.* RSC, WSS *v.* CSB14, *v.* CSB + *v.* no intervention		Facility-based	Weight gain, MUAC, length, WAZ, LAZ, WLZ, haemoglobin level	F
Huybregts, 2012	Cluster RCT	16	Chad (urban)	1038 (519)	6–36 months	RUSF *v.* no intervention	46 g/day (247 kcal/day)	Facility-based	Weight gain, MUAC, length, WAZ, LAZ, haemoglobin level, % anaemia	F
Karakochuk, 2012	Cluster RCT	16	Ethiopia (rural)	1125 (428)	6–60 months	CSB + vegetable oil *v.* RUSF	1413 kcal, 47 g protein (300 g CSB + 32 g oil) *v.* 500 kcal, 13 g protein (92 g)	Community-based	Weight gain, MUAC, length, WAZ, LAZ, WLZ, % recovery	F
Medoua, 2016	RCT	12	Cameroon (both)	81 (32)	6–59 months	CSB + *v.* RUSF	75 kcal/kg/day	Facility-based	Weight gain, MUAC, length, WLZ, WAZ, LAZ, % recovery, % death	F
Talavera, 2020	RCT	20	Mexico (rural)	682 (340)	24–60 months	RUSF *v.* non-RUSF	250 kcal	Community-based	Weight gain, length, WLZ, WAZ, LAZ	F

CSB++, corn-soy blend ‘plus plus’; LMF, locally milled flours + micronutrient powder; LNS, Lipid-based nutritional supplements; MI, Misola; RUSF, ready-to-use supplementary food; *v.*, versus; WSC, Extruded sorghum-cowpea FBFs white sorghum-cowpea; RSC, red sorghum-cowpea; WSS, A white sorghum-soy FBF; CSB14, An extruded corn-soy FBF, CSB+, A traditional non-extruded corn-soy FBF; NR, not reported; WLZ, weight-for-length *Z-*score; MUAC, mid-upper arm circumference; WAZ, weight-for-age *Z-*score, which is a composite of weight-for-height and height-for-age; LAZ, length-for-age *Z-*score; ICF, International classification of Functioning, Disability and Health; F, Function level, A, activity level, P, participation level.

### Outcome measures

All the outcome measures applied to determine the effectiveness of RUSF were at the body function and structure level of the ICF. To determine recovery from MAM, the cutoffs are WLZ > −1,^(43)^ WLZ > −2, and MUAC ≥ 12⋅5 cm.^(38)^ (Table 2). The anaemia status of the study participants was assessed based on the 2011 WHO definitions.^(40)^ In one study, recovery rates were assessed according to SPHERE target rates: >75 % for recovery, <15 % for defaults, and <3 % for deaths.^(41)^ Five studies defined nutritional recovery using WLZ > −2 as a primary outcome. measure,^(38–41,43)^ and three studies defined the haemoglobin level as a primary outcome measure^(38,40,42)^ Seven studies defined their primary outcomes as (1) the gain in weight, length, and MUAC, (2) the mean change in MUAC, WAZ (weight-for-age *Z*-score), LAZ (length-for-age *Z*-score), and WLZ (weight-for-length *Z*-score), (3) BMI,^(43)^ (4) the percentage of anaemia;^(38,42)^ and (5) the death rate^(39)^.

### Types of interventions

Table 2 shows the content of each intervention or dietary supplementation, the duration of the intervention, the place of intervention, and the ICF level classification of the outcomes.

The types of intervention used by different authors were RUSF, Corn-Soy Blend Plus Plus (CSB++), Misoloa (MI), and Locally milled flours + micronutrient powder (LMF)^(38)^, RUSF (75 kcal/kg/day),^(43)^ Extruded sorghum-cowpea FBFs (white sorghum-cowpea (WSC1), White sorghum-cowpea 2 (WSC2), red sorghum-cowpea (RSC)), a white sorghum-soy FBF (WSS), an extruded corn-soy FBF (CSB14), and a traditional non-extruded corn-soy FBF (CSB+),^(40)^ Supplementary Plumpy'Nut (Nutriset) (RUSF) was given biweekly for 16 weeks, and Corn/soy blend (CSB+) vegetable oil (premix) was given biweekly for 16 weeks,^(42)^ 300 g CSB and 32 g vegetable oil (1413 kcal, 47 g protein) or 92 g RUSF (500 kcal, 13 g protein) for 16 weeks,^(41)^ CSB+, RUSF (75 kcal/kg/day) was given every 2 weeks for 16 weeks,^(39)^ RUSF biscuits 250 kcal^(44)^.

### Effectiveness of RUSF compared to other dietary interventions or no intervention on anthropometric indices

**Fig. 2.**
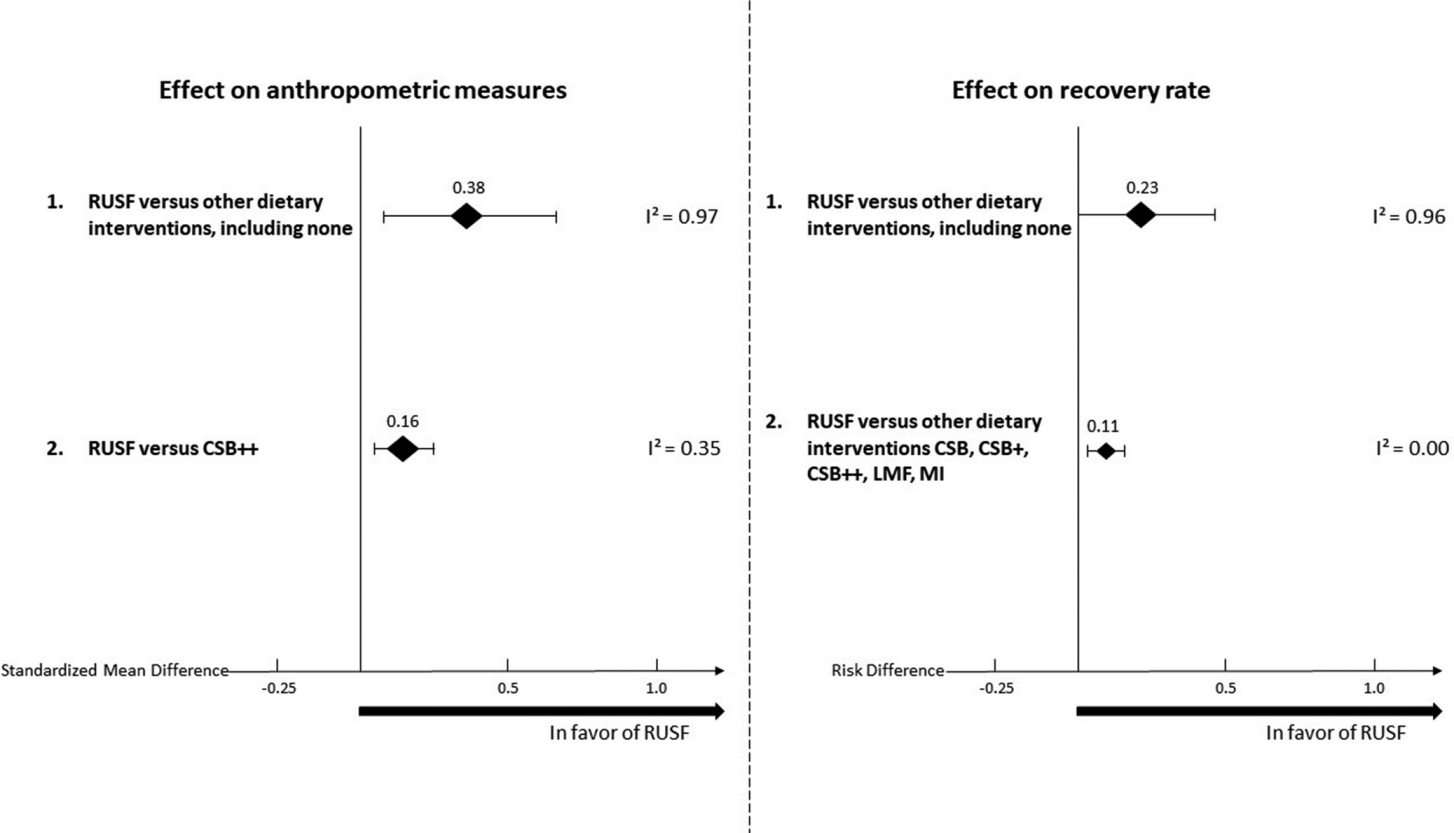
Summary of the pooled effect sizes (standardised mean differences and risk differences) of anthropometric measures and the recovery rate of RUSF compared to other interventions.

A summary of the pooled effect size of RUSF compared to other dietary interventions or no intervention on anthropometric indices is shown in Fig. 2. The raw data are presented in Table 3. Four studies (*n* 2880) compared RUSF supplementation to another dietary supplement (i.e. CSB++, LMF, MI, and control) or no intervention. The RUSF had a small but significant effect (pooled mean: 0⋅38; 95 % CI = [0⋅10, 0⋅67], *P* = 0⋅01) on different anthropometric measurements compared to the other supplements or no intervention in MAM children. However, there is too much heterogeneity among the included studies ( τ^2^ = 0⋅29; *χ*^2^ = 36⋅90; d.f. = 14 (*P* = 0⋅001); *I*^2^ = 97 %). This means that the observed variability in the included studies is most likely not due to chance but to diversity or differences between the studies themselves, suggesting the need for subclassification.

**Table 3.** Results at the individual study level

Reference	Groups	Weight gain (kg)		MUAC (cm)		Length (cm)		WLZ		LAZ		WAZ		Haemoglobin (dl)		% anaemia		% recovery	BMI		% death	Overall conclusion
		PRE	POST	PRE	POST	PRE	POST	PRE	POST	PRE	POST	PRE	POST	PRE	POST	PRE	POST		PRE	POST		
Ackatia-Armah, 2015 Azimi, 2020	RUSF (n 335)	7	8⋅2	11⋅9	13	70⋅8	73⋅7	−2⋅2	−1⋅3	2⋅5	NR			8⋅6	9⋅6	92⋅3	81⋅3	73⋅1 %				RUSF was more effective but more costly than other dietary supplements for the treatment of MAM; The RUSF group gained more in terms of weight, MUAC, and WLZ than those in the Misola and LMF groups, which did not differ from each other.
	CSB++ (n 342)	7⋅0	8⋅0	12	12⋅9	70⋅8	73⋅7	−2⋅3	−1⋅5	−2⋅3	NR			9⋅3	9⋅6	86⋅5	87⋅4	61⋅2 %				
	MI (n 306)	7⋅1	8⋅0	12	12⋅8	71⋅4	74⋅1	−2⋅3	−1⋅6	2⋅3	NR			9⋅2	9⋅1	87⋅9	91⋅9	61⋅1 %				
	LMF (n 281)	7⋅1	7⋅8	12	12⋅7	71	73⋅6	−2⋅3	−1⋅8	2⋅2	NR			8⋅7	9⋅3	91⋅6	88⋅6	57⋅9 %				
	RUSF (n 50)	11⋅5	12⋅9			93⋅0	94⋅4					−2⋅0	−0⋅8					92 %	13⋅3	14⋅5		A novel RUSF significantly improved growth indicators, and clinical symptoms of diarrhoea and fever in children with MAM.
	Control (n 49)	12⋅5	13⋅2			97⋅3	98⋅5					−1⋅9	−1⋅5					12⋅2 %	13⋅3	13⋅6		
Delimont, 2019	None (*n* 112)	12	13	14⋅7	14⋅8	87⋅6	90⋅5	−0⋅3	−0⋅2	−2	−1⋅8	−1⋅4	−1⋅2	10⋅1	10⋅8							The 24–53-month WSC and RSC group unadjusted WAZs were significantly greater than those in the WSC1 and WSS groups. The probability of WAZ deteriorating throughout the trial was 7–17 % across age groups.
	WSC2 (*n* 122)	12⋅4	13⋅5	14⋅9	15	88⋅1	91⋅9	−0⋅0	0⋅1	−2⋅1	−1⋅9	−1⋅2	−1⋅1	10	10⋅5							
	CSB14 (*n* 125)	12⋅2	12⋅9	14⋅7	14⋅8	86⋅4	89⋅8	−0⋅2	0⋅1	−2⋅3	−1⋅8	−1⋅3	−1⋅1	10⋅3	10⋅8							
	CSB + (*n* 117)	11⋅9	13	14⋅5	14⋅6	87⋅4	90⋅8			−2⋅1	−1⋅8	−1⋅4	−1⋅1	10⋅3	10⋅7							
	RSC (*n* 123)	12⋅6	13⋅4	15⋅1	14⋅9	89⋅3	91⋅8	−0⋅3	0⋅2	−1⋅7	−1⋅8	−1	−1⋅1	10⋅5	10⋅9							
	WSC1 (*n* 112)	11⋅9	12⋅8	14⋅5	14⋅5	87⋅5	91	−0⋅4	0⋅0	−2⋅2	−1⋅8	−1⋅5	−1⋅1	10⋅2	10⋅7							
	WSS (*n* 131)	12	12⋅7	14⋅3	14⋅3	86⋅9	90⋅3	−0⋅4	−0⋅1	−2⋅2	−1⋅9	−1⋅4	−1⋅1	10⋅4	10⋅9							
Huybregts, 2012	RUSF (*n* 598)	9⋅6	NR	14	14⋅3	80⋅1	NR			−1⋅7	−1⋅8	1⋅1	−1⋅1	10⋅4	10⋅6	61⋅9	56⋅5					RUSF significantly increased mean haemoglobin concentration at the end point by 3⋅8 g/l
	Control (*n* 440)	9⋅6	NR	14	14⋅1	79⋅9	NR			−1⋅9	−1⋅8	1⋅1	−1⋅1	10⋅4	10⋅3	61⋅5	66⋅7					
Karakochuk, 2012	RUSF (*n* 375)	9⋅3	NR	11⋅6	NR	85⋅7	NR	–2⋅6	NR	−1⋅9	NR	−3⋅1	NR					73 %				No significant differences between the RUSF and CSB groups; d and nonresponse rates were high (CSB group: 30 %; RUSF group: 24 %)
	CSB (*n* 750)	9⋅5	NR	11⋅7	NR	86⋅6	NR	–2⋅5	NR	−1⋅9	NR	−3⋅1	NR					67 %				
Medoua, 2016	CSB + (*n* 41)	8⋅3	10⋅1	12⋅9	13⋅0	80⋅7	NR	2⋅4	−1⋅6	−2⋅2	NR	−2⋅8	NR					73⋅2 %			0 %	The findings showed that both products were relatively successful in the treatment of MAM in children.
	RUSF (*n* 40)	7⋅9	10⋅6	13⋅0	13⋅1	79⋅5	NR	−2⋅4	−1⋅3	−2⋅0	NR	−2⋅6	NR					85 %			0 %	
Talavera, 2020	RUSF (*n* 377)	11⋅4	NR			90	NR	−1⋅4	NR	−1⋅9	−1⋅1	−2⋅0	−1⋅2									The MAM children who were treated with the RUSF group showed better WAZ and HAZ compared to the non-RUSF group.
	Non-RUSF (*n* 305)	12⋅1	NR			93	NR	−1⋅4	NR	−1⋅6	−1⋅1	−1⋅9	−1⋅3									

*Note*: RUSF, ready-to-use supplementary food, CSB, corn-soy blend; LNS, lipid-based nutrient supplement; MAM, moderate acute malnutrition; RCT, randomised controlled trial; WHZ, weight-for-height *Z-*score, MUAC, mid-upper arm circumference NR: no endline reported.

**Fig. 3.**
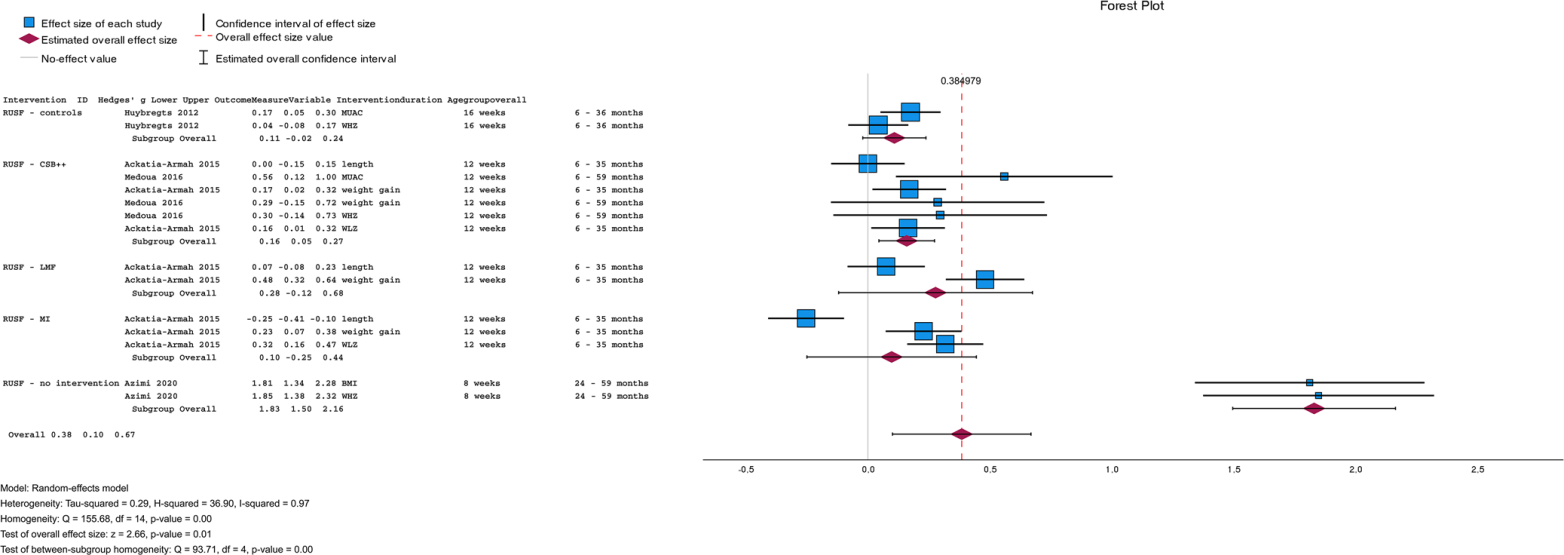
A random-effects meta-analysis comparing the mean difference of anthropometric measurements improved in children with MAM treated with a RUSF and other dietary supplementation types. Positive effect sizes indicate improvements in favour of the RUSF group, and negative effect sizes in favour of comparison.

Several trends were noticed by subgrouping per applied supplementary intervention. When MAM children received RUSF instead of LMF, they showed significantly more weight gain (*n* 1264, mean difference: 0⋅48; 95 % CI = [0⋅32, 0⋅64], *P* < 0⋅001 (Fig. 3). Two studies (*n* 1345) compared RUSF with CSB++ and showed that children treated with RUSF had a positive effect on their weight gain, MUAC, and WLZ/WHZ compared to those children treated with CSB++ (subgroup mean difference: 0⋅16; 95 % CI = [0⋅05, 0⋅27], *P* < 0⋅001). Children who were treated with MI showed a larger length change than the RUSF group (mean difference: −0⋅25; 95 % CI = [−0⋅41, −0⋅10], *P* < 0⋅001) (Fig. 3).

**Fig. 4.**
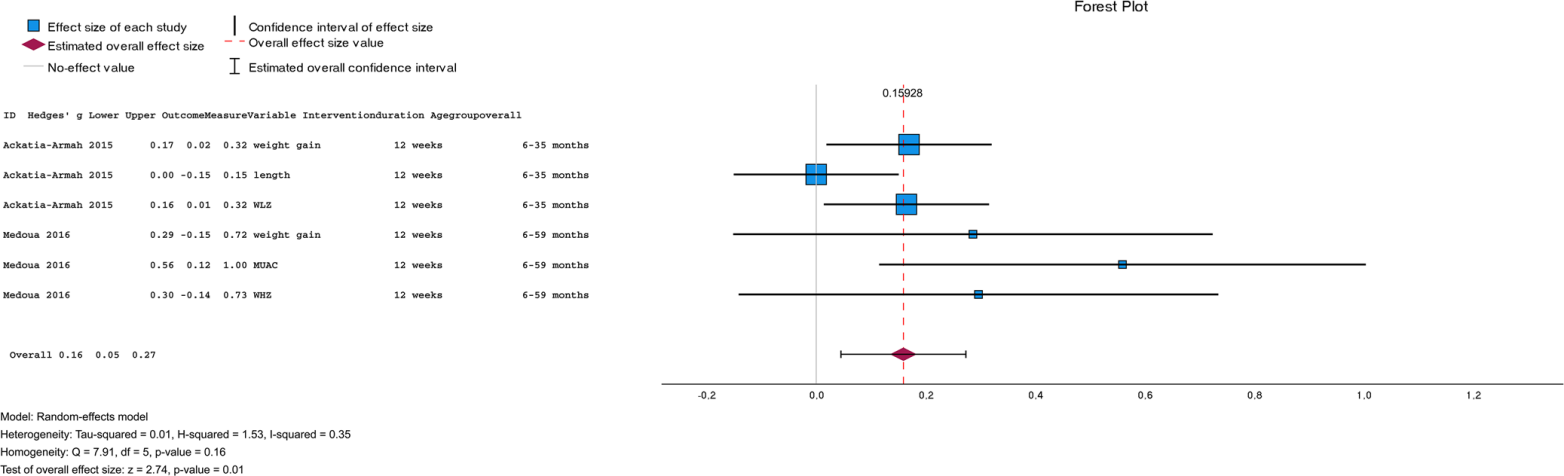
Subgroup meta-analysis comparing the mean difference of anthropometric measurements improved in children with MAM treated with a RUSF versus CSB++. Positive effect sizes indicate improvements in favour of the RUSF group, and negative effect sizes in favour of comparison.

A subgroup analysis (Fig. 2) using a random-effects meta-analysis of two studies (*n* 1345) comparing RUSF with CSB++, showed that children who were treated with RUSF had a small-sized but better effect on their weight gain, MUAC, and WLZ/WHZ compared to children who were treated with CSB++ (pooled mean: 0⋅16; 95 % CI = [0⋅05, 0⋅27], *P* = 0⋅01). There was no evidence of heterogeneity between those included studies (*I*^2^ = 35 %; *P* = 0⋅01) (Fig. 4).

### Effectiveness of the intervention in the recovery from MAM children

**Fig. 5.**
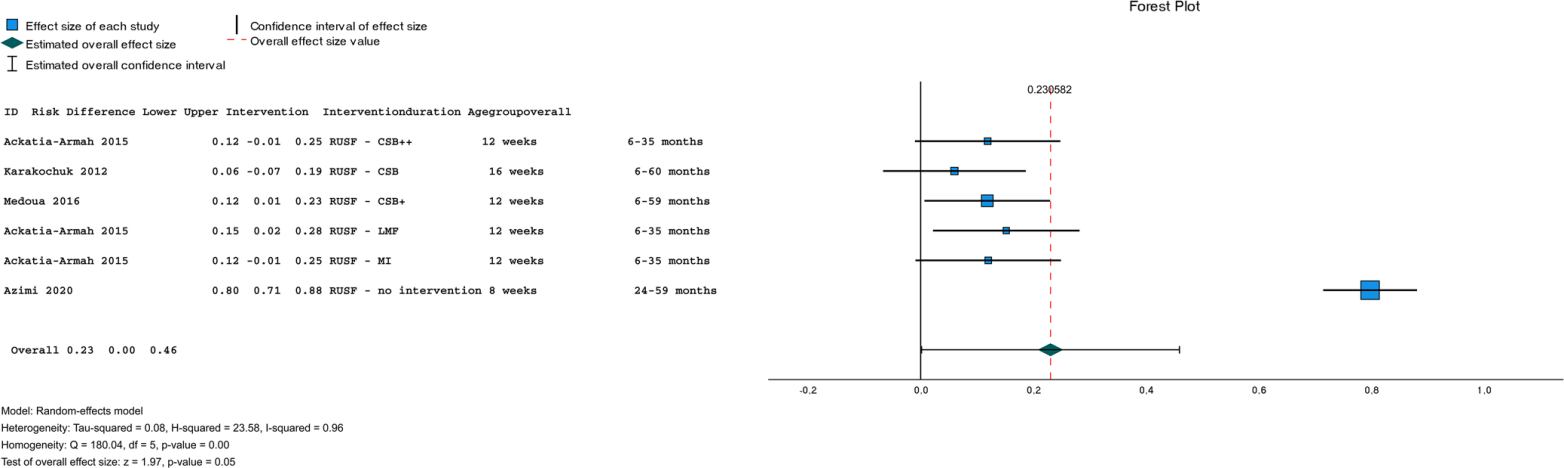
A random-effects meta-analysis of studies comparing the risk difference of children who recovered from MAM who were treated with a RUSF and other dietary supplementation types. Positive effect sizes indicate improvements in favour of the RUSF group, and negative effect sizes in favour of comparison.

A summary of the pooled effect size of RUSF compared to other dietary interventions or no intervention on the recovery rate is shown in Fig. 2. The raw data are presented in Table 3. Four studies (*n* 2570) investigated the recovery rate for MAM children who were treated with an RUSF and compared this to other types of dietary supplementation. The overall risk difference, when all interventions were pooled, was statistically significant (pooled risk difference: 0⋅23; 95 % CI = [0⋅00, 0⋅46], *P* = 0⋅05), and there was evidence of too much heterogeneity between the included studies (τ^2^ = 0⋅08; *χ*^2^ = 23⋅58, d.f. = 5 (*P* < 0⋅001); *I*^2^ = 96 %) (Fig. 5).

**Fig. 6.**
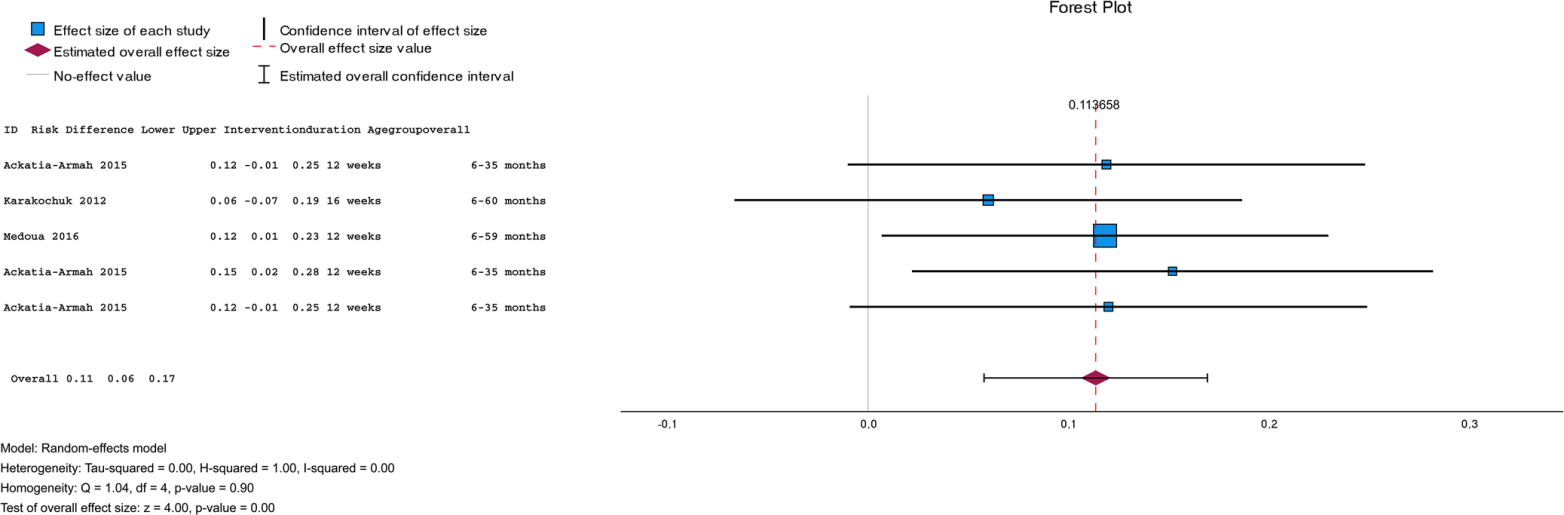
Subgroup meta-analysis of studies comparing the risk difference of children who recovered from MAM who were treated with a RUSF and other dietary supplementation types. Positive effect sizes indicate improvements in favour of the RUSF group, and negative effect sizes in favour of comparison.

The subgroup meta-analysis (Fig. 2) of three studies (*n* 2470) comparing RUSF to another type of supplementation (i.e. CSB, CSB++, LMF, and MI) showed that the risk differences in the recovery rate between MAM children who were treated with RUSF had a small advantage to recover compared to the other types of dietary supplementation (pooled risk difference: 0⋅11; 95 % CI = [0⋅06, 0⋅11], *P* < 0⋅001) and there was no significant heterogeneity between the included studies (*I*^2^ = 0⋅0 %, *P* < 0⋅001) (Fig. 6).

### Level of evidence

Overall, a low level of evidence was found for the effectiveness of RUSF on nutritional status and the recovery rate in MAM children due to some limitations (the risk of bias) and some imprecision. Further research is very likely to have an important impact on our confidence in the estimate of effect and is likely to change the estimate illustrated in Table 4.

**Table 4.** Quality of evidence appraised using the Grading of Recommendations, Assessment, Development, and Evaluation (GRADE) method

Study design: no. of studies, risk of bias (ref.)	Study limitations	Inconsistency of result	Indirectness of evidence	Imprecision	Quality of the evidence (GRADE)
RCT: 1 Moderate (Talavera *et al.*, 2020,), 2 High (Azimi *et al.*,2020, Medoua *et al.*, 2016)	Some limitations (↓)	Consistent (=)	Some indirectness (=)	Some imprecision (=)	⨁⨁◯◯
Cluster RCT: 2 Moderate (Huybregts *et al.*, 2012 and Karakochuk, *et al.*, 2012), 2 high (Ackatia-Armah *et al.*, 2015, and Delimont *et al.*, 2019)					

*Note*: ↓GRADE score downgraded by one point; = No impact on GRADE score; there was some uncertainty about downgrading the evidence because of indirectness. To compensate for the borderline decision, the precision category was not downgraded, despite some imprecision due to the small sample sizes of the included studies, as recommended by the GRADE guidelines.; ⊕ Point on final GRADE score awarded; ⊖ Point on final GRADE score not awarded and suggested representations of the quality of evidence; Symbol ⨁⨁⨁⨁ = high ⨁⨁⨁◯ = moderate ⨁⨁◯◯ = low, and ⨁◯◯◯ = very low.

RCT,  randomised controlled trial; cRCT, cluster-randomised controlled trial GRADE working group grades of evidence: High quality, we are very confident that the true effect lies close to that of the estimate of the effect; Moderate quality, further research is likely to have an important impact on our confidence in the estimate of effect and may change the estimate; Low quality, further research is very likely to have an important impact on our confidence in the estimate of effect and is likely to change the estimate; Very low quality, we are very uncertain about the estimate or we have very little confidence in the effect estimate; the true effect is likely to be substantially different from the estimate of effect.

## Discussion

This systematic review and meta-analysis aimed to investigate the effectiveness of RUSF compared to any other dietary supplementation intervention or no intervention on functioning at different levels of the ICF among children with MAM aged between 2 and 12 years old. The review summarises the findings from a total of seven studies, which include 6476 MAM children,^(36,38–44)^ aged between 6 months and 5 years. Despite the lack of strong evidence in the included literature, RUSF supplementation showed greater advantages in the improvement of nutritional anthropometric measurements (i.e. MUAC, weight gain, and WHZ) and recovery rate than the counter-comparative interventions, which is in line with previously conducted studies in infants.^(32)^^)^

In line with our results, a previously published meta-analysis in children under 5 years reported that dietary supplementation or food products showed a better recovery rate from MAM and improved anthropometric measurements compared to the control group;^(32)^ whey RUSF and local food products were comparable to the standard RUSF for recovery rate from MAM and weight gain, but the standard RUSF has better advantages compared to CSB.^(31)^ Although other types of supplementation have a positive effect on several anthropometric outcomes, that represent the children's undernutrition and/or recovery rate,^(45–47)^ RUSF has an even better effect. Similar to our findings in MAM children, Lenters and colleagues (2013),^(48)^ who reported on the overall effect of dietary interventions in children with MAM in their systematic review, concluded that MAM treated with RUSF had a significantly higher recovery rate, MUAC increase, and weight gain than those who received CSB.^(48)^ Yet, at that time, only five studies were available, mainly focusing on younger children.^(48)^ The present review adds that the nutritional status measurements of weight gain, MUAC, and WLZ/WHZ in MAM children who were treated with RUSF improved more than those receiving CSB++. The analyses also showed that children treated with Misola (MI) improved more concerning length gain than the RUSF group. This can be attributed to the content of the Misola, which contains a good mixture of soy, peanut kernel, and millet.^(49)^

Another important recurring finding in the literature^(48)^ that was confirmed in our study, is the higher recovery rate after treating MAM children with RUSF compared to other types of dietary supplementation. The recovery rate after RUSF varied in the included trials between 73 and 92 %,^(38,39,43)^ with slightly lower recovery results for soy/whey RUSF (67 %) and soy RUSF (59 %).^(41)^ Yet, the control interventions reached overall lower recovery rates (CSB variants: 62–73 %,^(39,41)^ Misola 61 %,^(38)^ Locally Middel Flours 57 %.^(38)^ Children with MAM require a higher intake of essential micro- and macronutrients than those required by healthy children.^(50)^ Soy/whey RUSF contains cocoa, making it tastier and more palatable than soy RUSF. Soy/whey RUSF, like soy RUSF, has a higher energy density and contains four times the quantity of animal source protein as CSB^++^.^(6,51)^ This may explain the beneficial effects of RUSF compared to other types of dietary interventions and therefore provide insights into wider avenues for policymakers, programme implementers, and policy decisions on the effectiveness of dietary interventions used to treat MAM.

### Strengths and limitations of this study

This systematic review and meta-analysis were conducted using a comprehensive search string that has been performed in three complementary databases without putting restrictions on the publication date. We did, however, limit ourselves to the English language. The intended age range is also limited to preschool and school-aged children. Nevertheless, we found no eligible studies on children older than five, which clearly emphasises a gap in the literature. Because of the paucity of eligible studies, no subgroup analysis could be performed on the different outcomes. Even though the forest plots (Figs. 3 and 4) seem to suggest a different representation of the results for weight gain, WHZ/WLZ in children aged between 6 and 35 months compared to those aged between 6 and 59 months old. Although the impact of RUSF seems larger in younger children for these outcomes, which has been confirmed in infants,^(52)^ too few studies are available to draw any conclusions about the impact of age on the effect of RUSF. Overall, the included studies posed a high risk of bias. Most of the research that met the eligibility criteria was conducted in an African country, which may affect the generalizability of the review. Nevertheless, this meta-analysis highlights the effectiveness of the promising results of RUSF in the treatment of MAM children and the need for more research on this vulnerable group.

However, the pooled estimates contain considerable levels of heterogeneity, both in terms of study design, and types of intervention, which are poorly captured between the included studies. Combining this with the rather poor methodological quality results in a poor level of evidence. Future studies and data are needed to evaluate the effectiveness of RUSF interventions to manage MAM and prevent its impact among children and pre-adolescents in developing countries. In particular, such studies should report on the pertinent outcome measures that do not only focus on nutrition-related areas, including body composition, muscle strength, anthropometric measurements, infections, and immunisation status but also capture functioning more holistically by measuring activity and participation levels in children with MAM of different age groups and different settings.

### Clinical implications and recommendations for future research

Currently, MAM children live in the community and do not receive standard treatment with supplementation. This meta-analysis provides insight into the current evidence for treating MAM children using RUSF. Children receiving RUSF exhibited better nutritional outcomes and recovery rates than children receiving another type of supplementation or no intervention at all. Despite its positive effects, the currently available evidence for RUSF is not sufficient. It is therefore not surprising that practical guidelines for the treatment of children with MAM do not exist yet. The WHO currently recommends children with MAM between ages 6–59 months old consume nutrient-dense food to meet their needs towards weight and height gain and functional recovery, and recommends supplementation only in cases of food insecurity and not routinely. The WHO does not specify any type of supplementation, does not make recommendations for older children,^(53)^ and emphasises that the currently available recommendations are not evidence-based. Methodologically stronger studies are therefore needed in the future. The effects of RUSF on outcomes beyond nutritional status and the recovery rate, such as psychomotor development and participation in daily activities, remain unidentified but are much needed. None of the included studies looked into the children's developmental features at a motor, cognitive, or social level, even though these issues have been established in these children in previous research.^(36,38–44)^ The link between the changes that occur in the nutritional status of MAM children and the type of supplementation or the changes at other levels of functioning and how they are intertwined needs to be disentangled in future research. Furthermore, supplements are given to strengthen the MAM children and empower them to participate in activities of daily life. As such, combined interventions focusing on both supplementation and physical stimulation may be of interest to help them gain an active lifestyle, thereby preventing the development of secondary adverse outcomes that arise from inactivity. Future research should include different age groups (i.e. children older than 5 years) and settings (rural or urban) of MAM children with RUSF interventions on functioning at different levels of the ICF among children with MAM above the age of five. It is well known that malnourishment negatively impacts overall functioning, including brain development, with peaks in its development beyond the age of five. As such, determining the impact of supplementation on these children is crucial as well, but has not been tackled yet. The dietary interventions provided, the types of intervention, design, duration of intervention, and outcome measurements are needed to illustrate the true potential of those dietary interventions. Long-term monitoring of infections and mortality, as well as assessment of BMI gain, weight gain, MUAC gain, body fat-free mass, muscle strength, motor skill competencies, and participation in daily activities and leisure, will better inform whether the RUSF product supplementation is associated with meaningful clinical advantages.

## Conclusion

This systematic review and meta-analysis show that RUSF interventions have a small-sized, but significantly better effect on the nutritional status and recovery rate of children with MAM compared to the use of CSB++. Future high-quality randomised control trials are needed to compare the effects of different types of dietary intervention on the different ICF components of children suffering from MAM. Specific attention should be given to different treatment methods and age groups, especially children over five.

## Supporting information

Teshome et al. supplementary material Teshome et al. supplementary material

## Supplementary material

The supplementary material for this article can be found at https://doi.org/10.1017/jns.2023.114
