# Supplementary material for: Current evidence on the effectiveness of Ready-to-Use Supplementary Foods in children with moderate acute malnutrition: a systematic review and meta-analysis
Source: J Nutr Sci. 2024 Jan 3;12:e130. doi: 10.1017/jns.2023.114 (PMC10765019; doi:10.1017/jns.2023.114)
Supplement: Teshome et al. supplementary material [file S2048679023001143sup001.docx]

**Appendix I**

**Search Strategy**

**I. PubMed** (**MEDLINE) search strategy (searched in title, abstract and/or keyword searches)**

(((("Child, Preschool" [Mesh] OR Child[Mesh] OR ((preschool[tw] OR school-aged[tw] OR school-age[tw] OR school[tw] OR under-5[tw] OR under-five[tw]) AND child*[tw]) OR toddler*[tw] OR preschool*[tw] OR kindergarten*[tw] OR "Pediatrics"[Mesh] OR Pediatric*[tw] OR "Under-5s"[tw] OR "Under 5"[tw] OR "Under-five"[tw])) AND (("Protein-Energy Malnutrition"[Mesh] OR "Protein-Energy Malnutrition"[Text Word] OR "Protein calorie malnutrition"[Text Word] OR (calorie[tw] AND protein[tw] AND energy[tw]) OR "Moderate Acute Malnutrition"[Text Word] OR "moderately acute malnourished"[Text Word] OR MAM[Text Word] OR "Wasting Syndrome"[Mesh] OR ((Moderate[Text Word] OR moderately[Text Word]) AND (acute[Text Word]) AND ("malnutrition"[MeSH Terms] OR "Malnutrition"[Text Word] OR "Undernutrition"[Text Word] OR "under-nutrition" [Text Word] OR "Malnourish*"[Text Word] OR "Undernourished"[Text Word] OR Wasted[Text Word] OR wasting[Text Word] OR "Starvation"[Mesh] OR Starvat*[Text Word] OR "Underweight"[Text Word] OR "Thinness"[Mesh] OR Thinness[Text Word] OR "Weight Loss"[Mesh] OR "Weight Loss"[Text Word] OR "Emaciation"[Mesh] OR Emaciation [Text Word])) OR "Nutrition Disorders" [Mesh] OR "Nutrition Disorders"[Text Word]))) AND ((("Nutrition Therapy"[Mesh] OR "Medical Nutrition Therapy"[Text Word] OR "Nutritional Support"[Mesh] OR "Nutritional Support"[Text Word] OR "Diet Therapy"[Mesh] OR "diet therapy"[Text Word] OR "diet therapies"[Text Word] OR "nutrition intervention"[Text Word] OR "Nutrition interventions"[Text Word] OR "nutrition program"[Text Word] OR "nutrition programs"[Text Word] OR "nutritional intervention"[Text Word] OR "Nutritional interventions"[Text Word] OR "nutritional program"[Text Word] OR "nutritional programs"[Text Word] OR "dietary intervention"[Text Word] OR "Dietary interventions"[Text Word] OR "dietary program"[Text Word] OR "dietary programs"[Text Word] OR "diet intervention"[Text Word] OR "Diet interventions"[Text Word] OR "diet program"[Text Word] OR "diet programs"[Text Word] OR "Dietary Supplements"[Mesh] OR "Dietary Supplements"[Text Word] OR "dietary supplement"[Text Word] OR "feeding intervention"[Text Word] OR "Feeding interventions"[Text Word] OR "feeding program"[Text Word] OR "feeding programs"[Text Word] OR "Targeted supplementary feeding"[Text Word] OR TSF[Text Word] OR "Blanket supplementary feeding"[Text Word] OR (Food[Mesh] OR "Food, Fortified"[Mesh] OR "Fortified Food"[Text Word] OR "Food, Formulated"[Mesh] OR "Foods, Specialized"[Mesh] OR "Fortified Food*"[Text Word] OR "Diet* Supplement*"[Text Word] OR "Food products"[Text Word] OR "Supplementary food"[Text Word] OR "Ready to use supplementary food"[Text Word] OR RUSF[Text Word] OR "Ready to use food*"[Text Word] OR RUF[Text Word] OR "Micronutrient* Supplement*"[Text Word] OR "Functional Food"[Mesh] OR "Functional Food"[Text Word] OR "Fortified blended food*"[Text Word] OR FBF [Text Word] OR "Corn soy* blend*"[Text Word] OR "Corn Soy Blend Plus"[Text Word] OR CSB++ [Text Word] OR "Wheat soy* blend*"[Text Word] OR "Super cereal plus"[Text Word] OR SC+[Text Word] OR "Corn-soy-whey blend"[Text Word] OR CSWB[Text Word] OR "Cereal pulse blend*"[Text Word] OR "Lipid-based nutrient supplement*"[Text Word] OR LNS[Text Word] OR "Nutributter"[Text Word] OR "Milk Proteins"[Mesh] OR "Milk Proteins "[Text Word] OR "Whey Proteins"[Mesh]) AND (therapy[Text Word] OR therapies[Text Word] OR intervention[Text Word] OR feeding[Text Word] OR interventions[Text Word] OR program[Text Word] OR Programs[Text Word] OR supplementation[Text Word] OR supplementations[Text Word])) OR "Community based therapeutic care"[Text Word] OR "Community-based management of malnutrition"[Text Word] OR CMAM[Text Word] OR "Community-based management"[Text Word] OR "Community-level interventions"[Text Word]))) AND ("Randomized controlled trials" OR RCT OR RCTs OR "Clinical Trial" OR "Controlled Clinical Trial" OR "quasi-randomized trials")

**II. Scopus search strategy (searched in title, abstract and/or keyword searches)**

( INDEXTERMS ( "child, preschool" ) OR INDEXTERMS ( child ) OR ( TITLE-ABS-KEY ( preschool OR school-aged OR school-age OR school OR under-5 OR under-five ) AND TITLE-ABS-KEY ( child* ) ) OR TITLE-ABS-KEY ( toddler* ) OR TITLE-ABS-KEY ( preschool* ) OR TITLE-ABS-KEY ( kindergarten* ) OR INDEXTERMS ( pediatrics ) OR TITLE-ABS-KEY ( pediatric* ) OR TITLE-ABS-KEY ( under-5s ) OR TITLE-ABS-KEY ( "under 5" ) OR TITLE-ABS-KEY ( under-five ) ) AND ( INDEXTERMS ( "protein-energy malnutrition" ) OR TITLE-ABS-KEY ( "protein-energy malnutrition" ) OR TITLE-ABS-KEY ( "protein calorie malnutrition" ) OR ( TITLE-ABS-KEY ( calorie AND protein AND energy ) ) OR TITLE-ABS-KEY ( "moderate acute malnutrition" ) OR TITLE-ABS-KEY ( "moderate acute malnourished" ) OR TITLE-ABS-KEY ( mam ) OR INDEXTERMS ( "wasting syndrome" ) OR ( ( TITLE-ABS-KEY ( moderate OR moderately ) AND acute ) ) AND INDEXTERMS ( "malnutrition" ) OR TITLE-ABS-KEY ( "malnutrition" ) OR TITLE-ABS-KEY ( "undernutrition" ) OR TITLE-ABS-KEY ( "under-nutrition" ) OR TITLE-ABS-KEY ( "malnourish*" ) OR TITLE-ABS-KEY ( "undernourished" ) OR TITLE-ABS-KEY ( wasted ) OR TITLE-ABS-KEY ( wasting ) OR INDEXTERMS ( "starvation" ) OR TITLE-ABS-KEY ( "starvat*" ) OR TITLE-ABS-KEY ( "underweight" ) OR INDEXTERMS ( "thinness" ) OR TITLE-ABS-KEY ( thinness ) OR INDEXTERMS ( "weight loss" ) OR TITLE-ABS-KEY ( "weight loss" ) OR INDEXTERMS ( "emaciation" ) OR TITLE-ABS-KEY ( emaciation ) OR INDEXTERMS ( "nutrition disorders" ) OR TITLE-ABS-KEY ( "nutrition disorders" ) ) AND ( INDEXTERMS ( "nutrition therapy" ) OR TITLE-ABS-KEY ( "medical nutrition therapy" ) OR INDEXTERMS ( "nutritional support" ) OR TITLE-ABS-KEY ( "nutritional support" ) OR INDEXTERMS ( "diet therapy" ) OR TITLE-ABS-KEY ( "diet therapy" ) OR TITLE-ABS-KEY ( "diet therapies" ) OR TITLE-ABS-KEY ( "nutrition intervention" ) OR TITLE-ABS-KEY ( "nutrition interventions" ) OR TITLE-ABS-KEY ( "nutrition program" ) OR TITLE-ABS-KEY ( "nutrition programs" ) OR TITLE-ABS-KEY ( "nutritional intervention" ) OR TITLE-ABS-KEY ( "nutritional interventions" ) OR TITLE-ABS-KEY ( "nutritional program" ) OR TITLE-ABS-KEY ( "nutritional programs" ) OR TITLE-ABS-KEY ( "dietary intervention" ) OR TITLE-ABS-KEY ( "dietary interventions" ) OR TITLE-ABS-KEY ( "dietary program" ) OR TITLE-ABS-KEY ( "dietary programs" ) OR TITLE-ABS-KEY ( "diet intervention" ) OR TITLE-ABS-KEY ( "diet interventions" ) OR TITLE-ABS-KEY ( "diet program" ) OR TITLE-ABS-KEY ( "diet programs" ) OR INDEXTERMS ( "dietary supplements" ) OR TITLE-ABS-KEY ( "dietary supplements" ) OR TITLE-ABS-KEY ( "dietary supplement" ) OR TITLE-ABS-KEY ( "feeding intervention" ) OR TITLE-ABS-KEY ( "feeding interventions" ) OR TITLE-ABS-KEY ( "feeding program" ) OR TITLE-ABS-KEY ( "feeding programs" ) OR TITLE-ABS-KEY ( "targeted supplementary feeding" ) OR TITLE-ABS-KEY ( tsf ) OR TITLE-ABS-KEY ( "blanket supplementary feeding" ) OR ( INDEXTERMS ( food ) OR INDEXTERMS ( "food, fortified" ) OR TITLE-ABS-KEY ( "fortified food" ) OR INDEXTERMS ( "food, formulated" ) OR INDEXTERMS ( "foods, specialized" ) OR TITLE-ABS-KEY ( "fortified food*" ) OR TITLE-ABS-KEY ( "diet* supplement*" ) OR TITLE-ABS-KEY ( "food products" ) OR TITLE-ABS-KEY ( "supplementary food" ) OR TITLE-ABS-KEY ( "ready to use supplementary food" ) OR TITLE-ABS-KEY ( rusf ) OR TITLE-ABS-KEY ( "ready to use food*" ) OR TITLE-ABS-KEY ( ruf ) OR TITLE-ABS-KEY ( "micronutrient* supplement*" ) OR INDEXTERMS ( "functional food" ) OR TITLE-ABS-KEY ( "functional food" ) OR TITLE-ABS-KEY ( "fortified blended food*" ) OR TITLE-ABS-KEY ( fbf ) OR TITLE-ABS-KEY ( "corn soy* blend*" ) OR TITLE-ABS-KEY ( "corn soy blend plus" ) OR TITLE-ABS-KEY ( csb++ ) OR TITLE-ABS-KEY ( "wheat soy* blend*" ) OR TITLE-ABS-KEY ( "super cereal plus" ) OR TITLE-ABS-KEY ( sc+ ) OR TITLE-ABS-KEY ( "corn-soy-whey blend" ) OR TITLE-ABS-KEY ( cswb ) OR TITLE-ABS-KEY ( "cereal pulse blend*" ) OR TITLE-ABS-KEY ( "lipid-based nutrient supplement*" ) OR TITLE-ABS-KEY ( lns ) OR TITLE-ABS-KEY ( "nutributter" ) OR INDEXTERMS ( "milk proteins" ) OR TITLE-ABS-KEY ( "milk proteins" ) OR INDEXTERMS ( "whey proteins" ) ) AND ( TITLE-ABS-KEY ( therapy ) OR TITLE-ABS-KEY ( therapies ) OR TITLE-ABS-KEY ( intervention ) OR TITLE-ABS-KEY ( feeding ) OR TITLE-ABS-KEY ( interventions ) OR TITLE-ABS-KEY ( program ) OR TITLE-ABS-KEY ( programs ) OR TITLE-ABS-KEY ( supplementation ) OR TITLE-ABS-KEY ( supplementations ) ) OR TITLE-ABS-KEY ( "community based therapeutic care" ) OR TITLE-ABS-KEY ( "community-based management of malnutrition" ) OR TITLE-ABS-KEY ( cmam ) OR TITLE-ABS-KEY ( "community-based management" ) OR TITLE-ABS-KEY ( "community-level interventions" ) ) AND ( TITLE-ABS-KEY ( "randomized controlled trials" ) OR TITLE-ABS-KEY ( rct ) OR TITLE-ABS-KEY ( rcts ) OR TITLE-ABS-KEY ( "clinical trial" ) OR TITLE-ABS-KEY ( "controlled clinical trial" ) OR TITLE-ABS-KEY ( "quasi-randomized trials" ) ) AND ( LIMIT-TO ( LANGUAGE , "english" ) )

**III. Web of science search strategy(searched in title, abstract and/or keyword searches)**

TS=("Child, Preschool" OR Child OR ((preschool OR “school-aged” OR “school-age” OR school OR under-5 OR under-five) AND child*) OR toddler* OR preschool* OR kindergarten* OR "Pediatrics" OR Pediatric* OR "Under-5s" OR "Under 5" OR "Under-five")) AND (TS=("Protein-Energy Malnutrition" OR "Protein calorie malnutrition" OR (calorie AND protein AND energy) OR "Moderate Acute Malnutrition" OR "moderately acute malnourished" OR MAM OR "Wasting Syndrome" OR ((Moderate OR moderately) AND (acute) AND ("malnutrition" OR "Undernutrition" OR “under-nutrition” OR “Malnourish*” OR “Undernourished” OR Wasted OR wasting OR Starvation OR Starvat* OR “Underweight” OR Thinness OR "Weight Loss" OR "Emaciation")) OR "Nutrition Disorders" OR “Nutrition Disorders”)) AND (TS=("Nutrition Therapy" OR "Medical Nutrition Therapy" OR "Nutritional Support" OR "Diet Therapy" OR “diet therapies” OR “nutrition intervention” OR "Nutrition interventions" OR “nutrition program” OR "nutrition programs" OR “nutritional intervention” OR "Nutritional interventions" OR “nutritional program” OR "nutritional programs" OR “dietary intervention” OR "Dietary interventions" OR “dietary program” OR "dietary programs" OR “diet intervention” OR "Diet interventions" OR “diet program” OR "diet programs" OR "Dietary Supplements" OR “dietary supplement” OR “feeding intervention” OR "Feeding interventions" OR “feeding program” OR "feeding programs" OR "Targeted supplementary feeding" OR TSF OR "Blanket supplementary feeding" OR ((Food OR "Food, Fortified" OR "Food, Formulated" OR "Foods, Specialized" OR "Fortified Food*" OR "Diet* Supplement*" OR "Food products" OR "Supplementary food" OR "Ready to use supplementary food" OR RUSF OR "Ready to use food*" OR RUF OR "Micronutrient* Supplement*" OR "Functional Food" OR "Fortified blended food*" OR FBF OR "Corn soy* blend*" OR "Corn Soy Blend Plus" OR CSB++ OR "Wheat soy* blend*" OR "Super cereal plus" OR SC+ OR "Corn-soy-whey blend" OR CSWB OR "Cereal pulse blend*" OR "Lipid-based nutrient supplement*" OR LNS OR "Nutributter" OR "Milk Proteins" OR "Whey Proteins") AND (therapy OR therapies OR intervention OR feeding OR interventions OR program OR Programs OR supplementation OR supplementations)) OR "Community based therapeutic care" OR "Community-based management of malnutrition" OR CMAM OR "Community-based management" OR "Community-level interventions" )) AND (TS=("Randomized controlled trials" OR RCT OR RCTs OR "Clinical Trial" OR "Controlled Clinical Trial" OR "quasi-randomized trials")) AND ( LIMIT-TO ( LANGUAGE , "English" ) )
